# Investigating the Impact of COVID-19 Vaccines on Liver Function: Insights From a Single-Institute Study

**DOI:** 10.7759/cureus.36588

**Published:** 2023-03-23

**Authors:** Rajmohan Rammohan, Melvin Joy, Tulika Saggar, Sai Greeshma Magam, Atul Sinha, Dilman Natt, Vaishali Mehta, Susan Bunting, Prachi Anand, Paul Mustacchia

**Affiliations:** 1 Gastroenterology and Hepatology, Nassau University Medical Center, East Meadow, USA; 2 Internal Medicine, Nassau University Medical Center, East Meadow, USA; 3 Pulmonary and Critical Care, Nassau University Medical Center, East Meadow, USA; 4 Rheumatology, Nassau University Medical Center, East Meadow, USA

**Keywords:** coronavirus disease 2019, covid-19, liver damage, liver function, covid-19 vaccine, elevated liver enzyme, covid 19

## Abstract

Introduction

SARS-CoV-2 can cause respiratory and extrapulmonary complications, including liver injury. Therefore, understanding the virus's impact on the liver and the coronavirus disease 2019 (COVID-19) vaccine's protective effect is crucial, given the correlation between hepatic involvement and disease severity. Our study aims to evaluate this relationship and the impact of vaccination on liver injury in COVID-19-infected patients.

Methods

A retrospective cohort study analyzed liver function outcomes in COVID-19-infected patients who received two doses of the Pfizer-BioNTech or Moderna mRNA vaccine from October 2019 to October 2021. The study population was matched based on baseline characteristics, and Fisher's T-test was used for analysis. Secondary outcomes included COVID-19-related death, hospital stay, and SARS-CoV-2 infection after the second dose. SPSS (IBM Corp., Armonk, NY) and RStudio (RStudio, PBC, Boston, USA) software were utilized to ensure robust statistical analysis.

Results

A group of 78 patients with a propensity score were matched and analyzed, resulting in two groups of 39 patients each: vaccinated and unvaccinated. The vaccinated group had a lower incidence of liver injury, reduced length of stay, and mortality. The study suggests that COVID-19 vaccination can positively impact infected patients. These findings should be considered when making decisions about vaccine distribution and usage, and more research is needed to fully understand the vaccine's impact on ending the pandemic.

Conclusion

This study emphasizes the COVID-19 vaccine's significance in reducing liver injury and related outcomes, such as length of stay and mortality in infected patients. The results provide further evidence of vaccination benefits, with implications for healthcare professionals and policymakers. Further research is needed to deepen our understanding of COVID-19's complex effects on the liver and the vaccine's impact. Investing in research can inform clinical management, improve patient outcomes, and ultimately help end the pandemic.

## Introduction

Severe acute respiratory syndrome coronavirus 2 (SARS-CoV-2) is the virus that caused the coronavirus disease 2019 (COVID-19) pandemic [1]. This virus has a range of respiratory and extrapulmonary complications, including liver injury [2], cardiac injury [3], gastrointestinal symptoms [4], neurologic symptoms [5], and kidney injury [6]. The liver plays a crucial role in detoxification and metabolism [7]; therefore, its involvement in SARS-CoV-2 infection can significantly impact treatment modalities. In addition, the liver's role in COVID-19 disease progression can range from asymptomatic transaminitis to acute liver failure [8].

The number of COVID-19-related death worldwide was 5.94 million [9], which has prompted a global effort to develop a vaccine to stop the pandemic. In light of the current global vaccination rate of 65% as of February 2023 [10], our study aims to understand the direct impact of SARS-CoV-2 on the liver and to determine if the COVID-19 vaccine provides any protective effect against liver injury. In addition, the relationship between elevated liver enzymes and vaccination status will be evaluated, as well as the impact of vaccination on liver injury in COVID-19-infected patients. Given the correlation between hepatic involvement and disease severity and the development of acute respiratory distress syndrome (ARDS) [10], understanding the vaccine's protective effect is critical.

Liver injury is a complex issue in COVID-19 patients and has multiple contributing factors. One of the leading causes is a direct infection of the liver cells (hepatocytes), which leads to toxic damage. Additionally, hypoxic reperfusion injury, drug-induced liver injury, hepatic congestion, and systemic inflammation that includes immunologic activation and cytokine storms can also cause liver injury [11]. Other factors that may result in abnormal liver biochemistries in COVID-19 patients include ischemic hepatitis, hepatic congestion linked to heart muscle disease (cardiomyopathy), and the release of transaminases due to the breakdown of skeletal and heart muscle [11].

Different forms of vaccines, including messenger ribonucleic acid (mRNA), protein subunit, and viral vector vaccines, have been produced by various manufacturers, each with its efficacy and dosing schedule [12]. These vaccines aim to prevent or reduce the severity of COVID-19 infection. Our study aimed to provide insight into the effectiveness of the COVID-19 vaccine in terms of liver function outcomes among infected patients and contribute to the growing body of knowledge on the impact of vaccination on COVID-19.

## Materials and methods

Study design

From October 2019 to October 2021, a retrospective cohort study was carried out to examine the outcomes of patients infected with COVID-19 who had been vaccinated and those who had not. The study population included patients who had either received two doses of the COVID-19 vaccine or remained unvaccinated. Both groups were given four doses of remdesivir 100 milligrams (mg) intravenously and five doses of Decadron 6 mg. To ensure an equitable comparison, the baseline characteristics of the two groups were matched before analyzing the results of the liver function test. The Fisher's T-test statistical method was utilized to compare the findings.

Data source

A dataset was gathered and analyzed using the Sunrise electronic medical record (EMR) software (AllScripts, Chicago, IL). It included diverse patient information like age, gender, comorbidities, COVID-19 vaccine status, SARS-CoV-2 infection status, hospitalization duration, morbidity, mortality, medication, vaccination date, time taken for infection from vaccination, basic metabolic labs, and liver function test outcomes.

Inclusion criteria

Our study population included adults aged 21 to 75 years who received two doses of either the Pfizer or Moderna vaccine. Only individuals who received a complete regimen of two doses were included in the study, and those who received only one dose were excluded. This selection criterion was established to ensure that the study captured a consistent level of vaccine protection among participants. The aim of including this specific age group and type of vaccine was to evaluate the effectiveness of these vaccines in providing immunity and reducing the risk of severe illness among this population.

Outcomes from the study cohort

The secondary outcomes included the time of COVID-19-related death, length of hospital stay, and the time of SARS-CoV-2 infection after the second dose of the vaccine. This information will contribute to the growing body of knowledge on the efficacy of COVID-19 vaccines in reducing the severity and duration of illness.

Reporting

In this study, both SPSS for Windows (IBM Corp., Armonk, NY) and RStudio software (RStudio, PBC, Boston, USA) were utilized to conduct a thorough and robust statistical analysis of the data collected. The use of multiple statistical tools ensured the accuracy and reliability of the results, providing a comprehensive understanding of the study outcomes. The analysis was aimed at interpreting the data and drawing meaningful conclusions from the findings. The results of the analysis will play a crucial role in informing future research on COVID-19 vaccination and its impact on health outcomes. The use of multiple statistical software programs is essential in research as it helps to mitigate potential biases and increase the validity and robustness of the results.

## Results

In this study, a group of 78 patients was selected and analyzed based on their propensity score after being matched. This resulted in two groups of 39 patients each, one group that was vaccinated and another that was unvaccinated. The average age of the patients was 58.4 years old, with a standard deviation of 13.3 and an equal representation of males and females in both groups. The average number of days between the last dose of the COVID-19 vaccine and infection was 132, with a standard deviation of 57. The patient's baseline characters are listed in Table 1.

**Table 1:** Patients' baseline characteristics Values are presented as numbers and mean as ± standard deviation.

	Vaccinated (n = 39)	Unvaccinated (n = 39)	P-value
Age	59.4 ± 12.3	57.4 ± 13.3	0.541
Sex, M/ F	18 (46%)/21 (53.8%)	17 (43.5%)/22 (56.4%)	0.656
Race			1.212
Hispanic	12 (30.7%)	13 (33.3%)	
Blacks	10 (25.6%)	11 (28.2%)	
White	9 (23.0%)	8 (34.7%)	
Others	8 (20.5%)	7 (17.9%)	
Hypertension (HTN)	12 (30.7%)	10 (25.6%)	0.751
Hyperlipidemia (HLD)	11 (28.2%)	12 (30.7%)	0.621
Chronic kidney disease (CKD)	3 (7%)	4 (10.2%)	0.801
Diabetes mellitus (DM)	9 (23%)	11 (28.2%)	1.048
Coronary artery disease (CAD)	6 (15.3%)	5 (12.8%)	0.655
Chronic obstructive pulmonary disease (COPD)	4 (10.2%)	6 (15.3%)	1.011
Asthma	3 (7%)	5 (12.8%)	1.021

According to the study findings, the group of patients who received the vaccine exhibited a significant decrease in the frequency of liver injury compared to the unvaccinated group. This deduction was made based on the outcomes of liver function tests, which demonstrated a lower average level of aspartate transaminase (AST) (42 ± 13 vs. 93.3 ± 35, P = 0.02), alanine transaminase (ALT) (50 ± 10 vs. 97.4 ± 45.5, P = 0.04), and alkaline phosphatase (ALP) (65 ± 15.2 vs. 93 ± 35.6, P < 0.01). Furthermore, the vaccinated patients had a shorter hospitalization period compared to the unvaccinated group (10.6 ± 4.8 vs. 18.1 ± 8.1, P = 0.042), as presented in Table 2.

**Table 2:** Comparison of liver enzyme levels and mortality rates in vaccinated and unvaccinated groups Values are presented as numbers and mean as ± standard deviation.

	Vaccinated group (n = 39)	Unvaccinated group (n = 39)	P-value
Aspartate transaminase (AST)	42 ± 13	93.3 ± 35	0.02
Alanine transaminase (ALT)	50 ± 10	97.4 ± 45.5	0.04
Alkaline phosphatase (ALP)	65 ± 15.2	93 ± 35.6	<0.01
Length of stay (LOS)	10.6 ± 4.8 (days)	18.1 ± 8.1 (days)	0.042
Mortality	8 (20.5%)	17 (43.5%)	0.031

The study also found that the vaccinated patient group had a lower mortality incidence than the unvaccinated group (8 vs. 17, P = 0.031). These results suggest that the COVID-19 vaccine can have a positive impact on reducing the incidence of liver injury, length of stay, and mortality in infected patients. This study adds to the growing body of evidence that supports the benefits of COVID-19 vaccination and will inform future research in this area.

The results of this study are significant as they provide further evidence of the positive impact that the COVID-19 vaccine can have on patients who are infected with the virus. By reducing the incidence of liver injury, length of stay, and mortality, the vaccine can help mitigate the pandemic's impact and improve the health outcomes of those affected.

## Discussion

Pathophysiology of COVID-19 causing transaminitis

The underlying pathophysiology of COVID-19 in the liver is complex, involving inflammation, decreased blood supply, and altered metabolic activity [13]. Therefore, understanding the mechanisms of liver toxicity induced by the virus, including direct viral invasion of hepatocytes, host inflammatory responses, and drug-induced liver injury from COVID-19 treatments, is crucial for developing effective treatments and informing clinical management [13].

SARS-CoV-2 enters the body through the respiratory tract and replicates in the epithelial cells of the lungs [14]. The virus can then spread to other organs, including the liver, where it can bind to angiotensin-converting enzyme 2 (ACE2) receptors and cause damage to liver cells [14]. The immune response to the virus can also lead to liver inflammation, resulting in liver dysfunction. Studies have shown that elevated levels of liver enzymes, such as ALT and AST, are typical in patients with COVID-19 and are predictive factors in intensive care unit (ICU) admissions [15].

In severe cases, the virus can cause thrombotic microangiopathy, reducing blood flow and leading to ischemia and cell death [15]. The virus can also affect the liver's metabolic functions, causing fatty liver disease, hyperglycemia, and decreased bile production. COVID-19-associated liver injury may present a worse outcome in patients with pre-existing liver disease, such as non-alcoholic fatty liver disease [16]. The use of antibiotics, nonsteroidal anti-inflammatory drugs (NSAIDs), and antiviral drugs in COVID-19 treatment can also have individual associations with liver injury [14]. The SARS-CoV-2 vaccine may protect against liver injury, as studies have shown that hepatic involvement in COVID-19 is linked to disease prognosis and ARDS [16]. The liver's role in the disease process of COVID-19 is still being studied, but it is clear that it plays a significant role in morbidity and mortality [15]. Therefore, further understanding of the underlying pathophysiology and mechanisms of liver toxicity can lead to the development of effective treatments and improved clinical management.

Patterns and frequency of liver biochemistry abnormalities in COVID-19

COVID-19 can lead to various liver-related issues, from minor transaminitis to severe acute liver failure. Transaminitis increases liver enzymes in the blood, which helps diagnose liver problems. Elevated levels of ALT, AST, gamma-glutamyl transferase (GGT), ALP, and total bilirubin (TBIL) can increase the risk of mortality [3]. Hospitalized COVID-19 patients have shown higher AST levels than ALT, which is unusual outside of specific conditions such as alcohol or drug-related liver damage, ischemic hepatitis, or cirrhosis [17]. The cause of this elevation is unclear but could be due to mitochondrial dysfunction, fatty liver, or changes in blood flow caused by microthrombotic disease. Studies show that 14-53% of COVID-19 patients may experience asymptomatic transaminitis, but 2-11% of severe cases can progress to acute or chronic liver disease, increasing the risk of death [18]. Those with severe COVID-19 have a higher rate of liver dysfunction and mortality rates than those without liver issues [17].

Clinical course and outcomes following SARS-CoV-2 infection in pre-existing chronic liver diseases

There are concerns that pre-existing chronic liver diseases, such as alcoholic liver disease, non-alcoholic fatty liver disease, cirrhosis, and hepatocellular carcinoma, can lead to poor outcomes in patients infected with SARS-CoV-2 [16]. People with chronic liver diseases often have immune system dysfunctions, including activated macrophages and impaired lymphocyte and neutrophil function, making them more susceptible to infections and severe inflammation [18]. Chronic liver disease also shares risk factors with severe COVID-19, including older age, male gender, and comorbidities such as hypertension, heart disease, diabetes, and cancer. Advanced liver disease can also cause coagulopathy and weaken the immune system, leading to more severe COVID-19 outcomes [19].

Vaccination against COVID-19

The COVID-19 pandemic has resulted in an unprecedented global effort to develop a vaccine to prevent its spread and mitigate its impact. The vaccines are designed to target the SARS-CoV-2 RNA virus at different stages of its life cycle, including binding to host cells, genomic release, translation into viral proteins, and replication [5]. The vaccines are available in different forms, such as mRNA vaccines, protein subunit vaccines, and viral vector vaccines, each with its own unique mechanism of action. In addition, various manufacturers have developed these vaccines, each with its own efficacy and scheduling [5].

The mRNA vaccines, such as the Pfizer-BioNTech and Moderna vaccines, use messenger RNA to instruct cells to produce a piece of the virus, which then triggers an immune response. The protein subunit vaccines, such as the Johnson & Johnson vaccine, use a piece of the viral protein to stimulate the immune system. Finally, viral vector vaccines, such as the AstraZeneca vaccine, use a harmless virus to deliver a piece of the viral genome into cells, triggering an immune response [20].

Each vaccine type has its own advantages and limitations, including varying efficacy and side effect profiles and different scheduling requirements. The goal of these vaccines is to prevent infection or reduce the severity of COVID-19 in those who are infected. The widespread distribution and administration of these vaccines have been a critical component of the global effort to control the pandemic and return to a sense of normalcy [21].

Effects of COVID-19 on the liver of vaccinated and unvaccinated patients

The liver is susceptible to the SARS-CoV-2 virus due to the presence of ACE2 receptors in biliary and liver epithelial cells [14]. These receptors provide a point of entry for the virus to cause damage to the organ, leading to elevated aminotransferases, which are most commonly seen in levels of AST and ALT, even in hospitalized patients without pre-existing liver diseases [22].

The impact of COVID-19 vaccinations on post-infection liver conditions has been studied with some noteworthy results. A study by Kulkarni et al. found that vaccinated patients who developed cholestasis after infection recovered faster with lower levels of ALP and glutamyl transpeptidase compared to unvaccinated patients who experienced prolonged cholestasis and a higher risk of needing a liver transplant [22]. Another study found that SARS-CoV-2 infection may trigger autoimmune or autoinflammatory dysregulation, leading to autoimmune hepatitis [2,23].

These findings underline the significance of early detection and treatment of post-COVID-19 liver conditions and the potential of vaccinations to improve outcomes. By altering the course of severe COVID-19 infections and reducing the risk of severe cholestasis, vaccinations may lower the risk of morbidity and death [22]. In a comparative study between the COVID-19 vaccine, breakthrough cases, and unvaccinated individuals, liver injury patterns and incidence were analyzed. The study revealed that partially vaccinated patients had elevated levels of ALT, AST, and bilirubin, while fully vaccinated individuals were not at risk of liver injury ​​​​​​[24]. Despite the rapid development of several COVID-19 vaccines, there is still concern about the virus's impact on the liver of vaccinated individuals [24].

Therefore, it is crucial to conduct further research to understand the possible long-term effects of SARS-CoV-2 on the liver, including the risk of liver failure and cirrhosis. Furthermore, it emphasizes the need for ongoing monitoring and research to determine the long-term impacts of SARS-CoV-2 on the liver and the possibility of developing virus-specific treatments to reduce the risk of liver damage in vaccinated individuals. Therefore, it is crucial for policymakers and healthcare professionals to consider these findings when making decisions about COVID-19 vaccine distribution and usage. As more research is conducted, we will have a better understanding of the full impact of the COVID-19 vaccine and its role in ending the pandemic.

One potential limitation of this study is its retrospective design, which may have introduced bias and confounding factors. Retrospective studies rely on previously collected data, which may need to be completed or more accurate, leading to potential errors in the analysis. Furthermore, the study only included patients who received the Pfizer-BioNTech or Moderna mRNA vaccines, limiting generalizability to other vaccine types. Additionally, the study population was relatively small, which may affect the statistical power and generalizability of the results. Nevertheless, the propensity score matching and statistical analysis have improved the study's robustness, enabling the assessment of liver function outcomes in COVID-19-infected patients who received mRNA vaccines. The results indicate that vaccination can positively impact infected patients, which has significant implications for healthcare professionals and policymakers. Finally, the study only focused on liver injury outcomes, and other COVID-19-related complications were not explored.

## Conclusions

In conclusion, this study highlights the significance of the COVID-19 vaccine in reducing the incidence of liver injury and its related outcomes, such as length of stay and mortality in infected patients. The results of this study provide further evidence of the benefits of COVID-19 vaccination and its positive impact on patient outcomes. The findings of this study have important implications for healthcare professionals and policymakers in their decision-making about COVID-19 vaccine distribution and usage.
